# Abundance and distribution of sylvatic dengue virus vectors in three different land cover types in Sarawak, Malaysian Borneo

**DOI:** 10.1186/s13071-017-2341-z

**Published:** 2017-08-31

**Authors:** Katherine I. Young, Stephanie Mundis, Steven G. Widen, Thomas G. Wood, Robert B. Tesh, Jane Cardosa, Nikos Vasilakis, David Perera, Kathryn A. Hanley

**Affiliations:** 1 Department of Biology, New Mexico State University, Las Cruces, NM USA; 2 Department of Biochemistry and Molecular Biology, University of Texas Medical Branch, Galveston, TX USA; 3 Department of Pathology and Center for Biodefense and Emerging Infectious Disease, Center for Tropical Diseases; Institute for Human Infections and Immunity, University of Texas Medical Branch, Galveston, TX USA; 4 Sentinext Therapeutics, Penang, Malaysia; 5 Institute of Health & Community Medicine, Universiti Malaysia Sarawak, Kota Samarahan, Sarawak Malaysia

**Keywords:** *Aedes*, Mosquito, Spillover, Sylvatic, Dengue virus, Arbovirus, Land cover, Borneo

## Abstract

**Background:**

Mosquito-borne dengue virus (DENV) is maintained in a sylvatic, enzootic cycle of transmission between canopy-dwelling non-human primates and *Aedes* mosquitoes in Borneo. Sylvatic DENV can spill over into humans living in proximity to forest foci of transmission, in some cases resulting in severe dengue disease. The most likely vectors of such spillover (bridge vectors) in Borneo are *Ae. albopictus* and *Ae. niveus*. Borneo is currently experiencing extensive forest clearance. To gauge the effect of this change in forest cover on the likelihood of sylvatic DENV spillover, it is first necessary to characterize the distribution of bridge vectors in different land cover types. In the current study, we hypothesized that *Ae. niveus* and *Ae. albopictus* would show significantly different distributions in different land cover types; specifically, we predicted that *Ae. niveus* would be most abundant in forests whereas *Ae. albopictus* would have a more even distribution in the landscape.

**Results:**

Mosquitoes were collected from a total of 15 sites using gravid traps and a backpack aspirator around Kampong Puruh Karu, Sarawak, Malaysian Borneo, where sylvatic DENV spillover has been documented. A total of 2447 mosquitoes comprising 10 genera and 4 species of *Aedes*, were collected over the three years, 2013, 2014 and 2016, in the three major land cover types in the area, homestead, agriculture and forest. Mosquitoes were identified morphologically, pooled by species and gender, homogenized, and subject to DNA barcoding of each *Aedes* species and to arbovirus screening. As predicted, *Ae. niveus* was found almost exclusively in forests whereas *Ae. albopictus* was collected in all land cover types. *Aedes albopictus* was significantly (*P* = 0.04) more abundant in agricultural fields than forests. Sylvatic DENV was not detected in any *Aedes* mosquito pools, however genomes of 14 viruses were detected using next generation sequencing.

**Conclusions:**

Land cover type affects the abundance and distribution of the most likely bridge vectors of sylvatic DENV in Malaysia Borneo. Conversion of forests to agriculture will likely decrease the range and abundance of *Ae. niveus* but enhance the abundance of *Ae. albopictus*.

**Electronic supplementary material:**

The online version of this article (10.1186/s13071-017-2341-z) contains supplementary material, which is available to authorized users.

## Background

The four serotypes of mosquito-borne dengue virus (DENV-1-4), the etiological agents of dengue fever and dengue hemorrhagic fever/shock syndrome, are transmitted among humans by *Aedes aegypti* across the tropical and subtropical regions of the world [1]. In the 1950’s, Smith proposed the existence of a sylvatic, enzootic cycle of DENV when he discovered high seroprevalence of anti-DENV antibodies in rural human populations in Malaysia in areas where *Ae. aegypti* were absent [2, 3]. *Aedes albopictus* were abundant in these areas, suggesting that this species might act as a bridge vector between an enzootic reservoir of DENV and humans [2, 3]. *Aedes albopictus* is a tree-hole breeding mosquito that adapts easily to a wide variety of environments including cities [4]. This species prefers to feed on humans but will feed opportunistically on a wide variety of non-human animals in proportion to their relative abundance in the environment [4]. *Aedes albopictus* is well known as a secondary vector for human-endemic DENV [5] and is susceptible to sylvatic DENV in laboratory studies (Mayer, Hanley and Vasilakis, unpublished data).

Subsequent to Smith’s studies, Rudnick conducted systematic studies of the ecology of sylvatic DENV in Malaysia in which he detected anti-DENV antibodies in canopy-living primates and isolated DENV from sentinel monkeys placed in the canopy [6]. Additionally, he collected approximately one million mosquitoes belonging to 300 different species and screened them for DENV [6]. However, he isolated sylvatic DENV only once, from a single pool of *Ae. niveus. Aedes niveus* comprises an arboreal species complex of at least 30 individual species [7]. In Rudnick’s study *Ae. niveus* were collected almost exclusively in monkey-baited traps, demonstrating the primatophilicity of this species [6]. Subsequent phylogenetic analyses of the sylvatic DENV isolates collected by Rudnick and by others in West Africa have revealed that each of the four human-endemic serotypes of DENV emerged independently from the Asian sylvatic cycle, demonstrating the propensity of sylvatic DENV for emergence [8, 9].

**Fig. 1:**
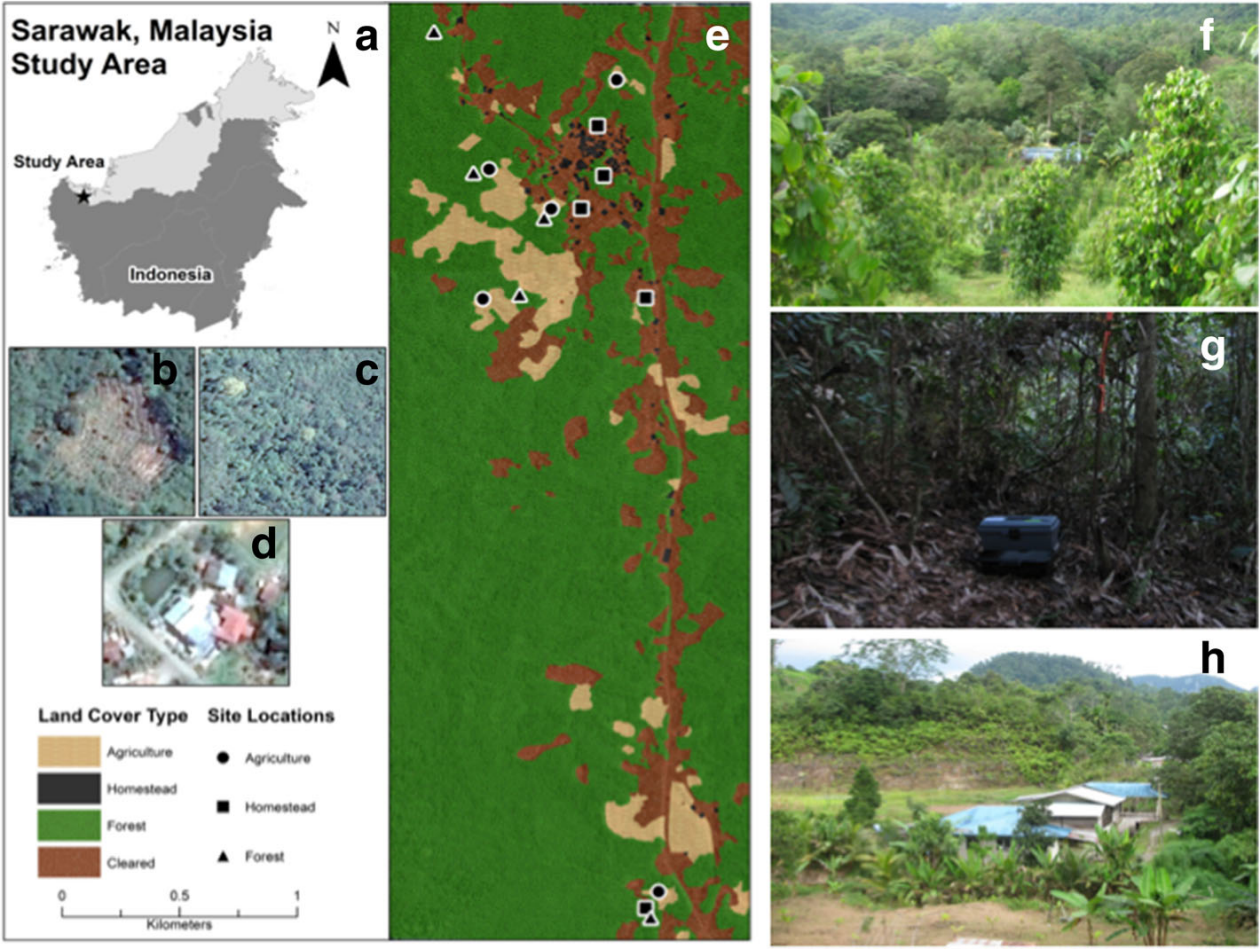
**a** A map of Borneo; the star indicates the study location. **b-d** Examples of images of satellite imagery used for land cover classification of agriculture (**b**), forest (**c**) and homestead (**d**) (ArcMap 10.2, ESRI, Redlands, California). **e** Overview of land classification at the study site with each sampling site indicated; **f-h** from top to bottom, images of agriculture, forest and homestead

Rudnick’s studies ended in the late 1970’s and since then Asia has existed in a “surveillance vacuum” [10] with respect to sylvatic DENV. Within the last decade, sylvatic DENV has been isolated four times from patients infected in Asia, one in peninsular Malaysia and three in Borneo (Fig. 1). Notably, all four experienced disease, which was severe in at least three of the patients. The first patient, a 20 year-old male who had recently been on holiday in peninsular Malaysia near one of Rudnick’s forest study sites, presented with clinical dengue hemorrhagic fever in 2008 [11]. Phylogenetic analyses determined the virus, DKD-811, to be a sylvatic DENV-2 most closely related to a sylvatic DENV-2 virus isolated by Rudnick in 1970 [11]. The second patient, a 37-year-old farmer, was admitted to hospital in 2007 with suspected dengue fever and warning signs of DHF (Cardosa, personal communication). Importantly, prior to infection this patient had been assisting with the clearance of forest in support of building a hydro-electric dam (Cardosa, personal communication). Through phylogenetic analysis, the virus isolate, DKE-121, was shown to be of sylvatic origin; however, it is antigenically distinct and genetically divergent from sylvatic DENV 1–4 (Vasilakis, personal communication). Sylvatic DENV-1 was isolated from an Australian researcher visiting the rainforest of Brunei [12]. The patient had returned to her hometown of Brisbane, Australia and presented with clinical dengue disease. Phylogenetic analysis of the virus isolate, Brun2014, showed the isolate to be a sylvatic DENV-1 that was highly distinct from other DENV-1 isolates [12]. Most recently, an extremely ancestral strain of DENV-2, QML22/2015, was isolated from an Australian tourist after returning home from Borneo. Interestingly, this isolate is ancestral to both sylvatic and human DENV-2 isolates, but more closely aligns with the sylvatic DENV-2 strains and did not react with non-sylvatic DENV-2 monoclonal antibodies [13].

Together, these ecological and clinical studies of sylvatic DENV suggest that human movement into the forest as well as land use changes that increase human contact with the forest may facilitate spillover into humans, with the potential to launch novel DENV strains or serotypes into a human-endemic cycle. At present, Malaysian Borneo is undergoing intensive and accelerating forest clearance [14–16]. In the current study, we sought to gauge the potential consequences of ongoing changes in land cover for sylvatic DENV spillover by characterizing the distribution of *Ae. albopictus* and *Ae. niveus* in the three predominant land cover types, forests, homesteads and agricultural fields. Collection sites were proximate to Kampong Puruh Karu, the site of spillover of DENV DKE-121. We hypothesized that *Ae. niveus* and *Ae. albopictus* would show significantly different distributions among these land cover types and predicted that: (i) *Ae. niveus* would be most abundant in forests, due to its preference for canopy habitat and its primatophilic feeding behavior [6]; (ii) in the other two land cover types, *Ae. niveus* would be more abundant at forest edges, following the logic of the first prediction, and (iii) compared to *Ae. niveus*, *Ae. albopictus* would be more evenly distributed in all land cover types. Additionally, we hypothesized that land cover type would affect the abundance and distribution of arboviruses detected in resident mosquitoes.

## Methods

### Study area

**Fig. 2:**
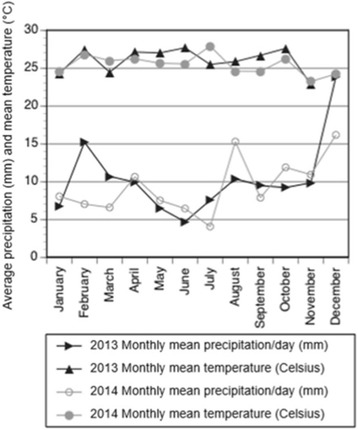
The average monthly precipitation (lower lines) and mean monthly temperature (upper lines) for the study location in Sarawak, Malaysian Borneo for 2013 (dark lines) and 2014 (light lines) (MODIS, http://modis.gsfc.nasa.gov/)

The study was conducted in and around Kampung Puruh Karu (1°15′19.09″N, 110°16′58.61″E), approximately 43 km southeast of Kuching city in Sarawak, Malaysian Borneo (Fig. 1). The climate of Borneo is typified by warm temperatures, averaging between 25 and 30 °C, high relative humidity (typically over 70%) and rainfall throughout the year (Fig. 2). The study area is predominately composed of rural communities surrounded by agricultural fields and forest patches. Communities comprise homesteads where extended families live together in separate households and maintain small gardens including fruiting trees, small rice paddies, and other agricultural crops. Most homesteads also own separate agricultural plots away from the community where they conduct large scale farming of either sustenance or economic crops. These latter plots have mainly been created by slash and burn methods within forests close to the community. According to local people, most of the forested area surrounding Kampung Puruh Karu had been cleared within the last twenty to thirty years, and these forests are now dominated by several different bamboo species, dipterocarp trees, fruiting trees, and tall grasses.

From September to December of 2013 and from June to July 2014, we collected mosquitoes in 15 sampling sites distributed among three land cover types (Fig. 1): 5 homestead, 5 agricultural and 5 forest sites. A collection and research permit, Sarawak Forestry Research permit number NCCD 907 4 4(9)–29, was obtained for the area and duration of the study. We collected in 3 sites within each land cover in 2013 and 2 sites within each land cover in 2014. We defined homesteads as any group of habitations where villagers lived for extended periods of time. Homes built within agricultural fields were not considered homesteads, as they were only inhabited briefly during planting, maintaining and harvesting of crops. Agriculture land cover was defined as any matrix of contiguous agricultural crops. The matrix of each agriculture site was not homogenous as crops ranged from mixed crops to several single crops depending on the needs of the field owners. Forest sites were defined as any location with consistent canopy cover. Table 1 provides a description of each sampling site. During April and May of 2016, the same 15 sampling locations were briefly resampled. Notably, 2 forests (FSTAT and FSTST) and 1 farm (FRMCO) had been altered since 2014. Forest site FSTAT had been completely cleared by the slash and burn method and no new growth had begun. Forest site FSTST had also been largely altered, with much of the area completely cleared by cutting as well as the use of herbicide; although, at this site some large fruiting trees and ground vegetation remained. The agriculture site FRMCO had been converted from entirely black pepper to a mix of agricultural crops consisting largely of banana plants.

**Table 1:** Mosquito trapping locations, including trapping month and year and major land cover characteristics for 15 sampling locations in three land cover types

Land cover type/sites	Latitude	Longitude	Elevation (m)	Trapping month/year	Surrounding area
Homestead: Human settlements in a rural area with small subsistence farming surrounding homes					
HOMJD	1°17′03.24″N	110°16′52.76″E	39.940	October 2013	Homes (3), domestic animals, fruiting trees, small gardens
HOMAT	1°16′58.55″N	110°16′49.69″E	39.179	November 2013	Home (1), fruiting trees, small gardens
HOMST	1°15′19.09″N	110°16′58.61″E	35.456	November 2013	Home (1), fruiting trees, forest
HOMVS	1°17′10.34″N	110° 16′51.95″E	39.929	June 2014	Home (1), domestic animals, small gardens
HOMMR	1°16′45.92″N	110°16′58.59″E	46.025	June 2014	Home (1), domestic animals, fruiting trees, small gardens
Agriculture: Matrix of agricultural crops with no permanent households					
FRMJD	1°16′45.75″N	110°16′36.17″E	63.055	October 2013	Forest edge, large scale subsistence agriculture, cocoa trees
FRMAT	1°16′58.52″N	110°16′45.58″E	49.481	November 2013	Forest edge, black pepper agriculture
FRMST	1°15′21.44″N	110°17′00.39″E	54.969	November 2013	Forest edge, black pepper agriculture
FRMLR	1°17′16.83″N	110°16′54.60″E	36.271	July 2014	Large scale subsistence agriculture, cocoa trees
FRMCO	1°17′04.15″N	110°16′37.03″E	56.693	July 2014	Forest edge, black pepper agriculture
Forest: Primarily secondary forest growth with consistent canopy cover					
FSTJD	1°16′46.30″N	110°16′41.21″E	69.494	December 2013	Consistent forest canopy, large fruiting trees, some secondary forest growth
FSTAT	1°16′57.15″N	110°16′44.58″E	64.008	December 2013	Consistent forest canopy, secondary forest growth
FSTST	1° 15′17.69″N	110°16′59.29″E	66.747	November 2013	Thick forest canopy, large fruiting trees, little disturbance
FSTHT	1°17′23.59″N	110°16′29.39″E	71.933	July 2014	Thick forest canopy, large fruiting trees, little disturbance
FSTCO	1°17′03.60″N	110°16′34.83″E	70.714	July 2014	Consistent forest canopy, large fruiting trees, some secondary forest growth

### Climate data and land cover delineation

Data from the Tropical Rainfall Measuring Mission (TRMM) 3B42 V7 product and the level-3 Moderate Resolution Imaging Spectroradiometer (MODIS) Land Surface Temperature (LST) and Emissivity A2 product were used to compare annual precipitation and temperature patterns for 2013 and 2014. Daily-accumulated rainfall measures from TRMM for a central location within the study area (1°17′03.00″N, 110°16′52.00″E) were used to generate estimates of monthly averages of daily rainfall for the study area. The MODIS LST data were retrieved as averaged values of daytime clear-sky LST for 8-day periods at a 1-km resolution covering the study area. Data from 8-day periods with >50% missing pixels due to cloud cover were excluded. The 8-day average temperatures were then used to generate approximate monthly average temperatures for the study area. To compare the patterns observed for these two years to the normal trends in the area, we included data on average temperature and precipitation for the study area from WorldClim interpolated climate layers, which show averages from 1950 to 2000 at 1 km spatial resolution.

The land cover map was created through on-screen digitizing in ArcMap 10.2, using Google Earth imagery from April 22nd, 2014. The three land cover types were assessed visually and delineated. Areas that appeared to be either bare earth or non-forested vegetation were classified as “Cleared.”

### Mosquito collection

Mosquito sampling was conducted using both gravid traps (Bioquip, Rancho Dominguez, California, USA) and a backpack aspirator (Bioquip). The order of sampling each site was haphazard as sampling could only commence after permission was granted from landowners. Collections were taken for 3 consecutive days at a given sampling site. Gravid traps were run continuously over these 3 days and samples were collected daily from 9 gravid traps per site at 08:00 and 16:00 h. Temperature at the time of collection was recorded for each trap. Backpack collections were performed twice daily at 08:00 and 16:00 h at each of the gravid trap locations. The backpack was run for 5 min while standing next to the trap location and moving the collection receptacle through the air, across vegetation if near the trap location and around the body of the collector.

Nine traps of each type were placed at each site; one trap at the centroid of the site, and two traps along four 90 degree transects: one half way between the centroid and the perimeter and one at the intersection of each transect and the perimeter. Mosquitoes were removed from each trap and anaesthetized using chloroform. Individual mosquitoes were identified morphologically to genus and, when possible, species, using taxonomic keys [17–19] and photographed. Samples were then pooled (< 40 mosquitoes/pool) by location, trap type, date collected, genus, known or putative species group (i.e. *Aedes* sp. A for a putative *Aedes* species), and sex. The pooled samples were held in liquid nitrogen until transported to the laboratory at the Institute of Health and Community Medicine at the Universiti Malaysia Sarawak (UNIMAS) where they were stored at -80 °C. All sampling locations were briefly resampled in April and May of 2016 using the backpack aspirator methods described above; however, each location was only sampled for 1 day rather than 3 consecutive days. Mosquito identification during resampling was based solely on morphological examination using available taxonomic keys; as well as the WRBU online interactive dichotomous key and unpublished reference guides made by the Sarawak Vector Control Department [7, 17–19].

### Virus screening

At UNIMAS, mosquito pools were homogenized by bead beating for 3 min at 26 rounds per min (rpm) in 1 ml of prepared stock homogenization media (500 ml DMEM hi-glucose (Gibco, Pittsburgh, Pennsylvania, USA) supplemented with 2.5 μg/ml amphotericin B (Gibco) 10% fetal bovine serum (FBS) (Gibco) and 1× penicillin-streptomycin mixture (Gibco). After homogenization, samples were centrifuged for 5 min at 8000 rpm, and 400 μl of supernatant was passed through a 0.25 μm syringe filter and stored at -80 °C until screened for arboviruses. Samples were then shipped on dry ice to University of Texas Medical Branch (UTMB) for arbovirus assay, after which the remaining homogenate was returned to -80 °C and shipped to New Mexico State University (NMSU) for DNA barcoding.

At UTMB, 100 μl of each filtered mosquito homogenate was inoculated into 12.5 cm^2^ culture flasks of African green monkey kidney (Vero) or *Ae. albopictus* (C6/36) cells. Vero cell cultures were held for 14 days at 37 °C and examined regularly for evidence of viral cytopathic effect (CPE). The C6/36 cell cultures were incubated at 28 °C for 7 days and observed daily for CPE. If CPE was observed, the cell culture supernatant was collected from the flask, clarified by centrifugation at 3200 rpm for 5 min, and transferred to a clean 1.5 ml Eppendorf tube. Total RNA was then extracted from an aliquot of the clarified cell culture supernatant through the Trizol method and resuspended in 50 μl RNase/DNase and protease-free water (Ambion, Austin, Texas). Additional aliquots were stored at -80 °C to attempt isolation of viruses from samples revealed to contain viral genomes by next-generation sequencing. Sequencing for viral screening was conducted on 14 CPE positive *Aedes* pools total.

### Next-generation sequencing to detect viruses

RNA (0.1–0.2 μg) quantified by NanoDrop 1000 spectrophotometer (Thermo Fisher Scientific, Pittsburgh, Pennsylvania, USA), was fragmented by incubation at 94 °C for 8 min in 19.5 μl of fragmentation buffer (Illumina, San Diego, California, USA). Following fragmentation, first and second strand synthesis, adapter ligation and amplification of the library were performed using the TruSeq RNA Library Prep Kit v2 under conditions prescribed by the manufacturer (Illumina). The samples were tracked using the index tags incorporated into the adapters as defined by the manufacturer.

Cluster formation of the library DNA templates was performed with the Illumina TruSeq PE Cluster Kit v3 and the Illumina cBot workstation using conditions recommended by the manufacturer. Paired end 50 base sequencing by synthesis was performed using Illumina TruSeq SBS kit v3 on an Illumina HiSeq 1000 using protocols defined by the manufacturer. Cluster density per lane was 645–980 k/mm^2^ and post-filter reads ranged from 148 to 178 million per lane. Base call conversion to sequence reads was performed using CASAVA-1.8.2. The *de novo* assembly program ABySS [20] was used to assemble the reads into contigs, using several different sets of reads, and k values from 20 to 40. In certain cases, pre-filtering of host-derived reads by mapping to *Ae. albopictus* and *Ae. aegypti* reference sequences enhanced the assembly process. Reads were mapped back to the contigs using bowtie2 [21] and visualized with the Integrated Genomics Viewer [22] to verify that the assembled contigs were correct.

### Mosquito DNA barcoding

Mosquito homogenates were subject to mitochondrial cytochrome *c* oxidase subunit 1 (*cox*1) barcoding at NMSU. The steel bead was removed from individual pools by magnet and each sample was centrifuged at 13,000× *g* for 5 min at 4 °C. The supernatant was removed and discarded; total DNA and RNA were extracted from the remaining homogenates using Trizol reagent (Invitrogen, Pittsburgh, Pennsylvania) according to the manufacturer’s protocol. The resulting DNA and RNA pellets were air-dried for up to 1 h and reconstituted in 10 μl of DEPC treated nuclease free water (Invitrogen).

The *cox*1 gene region has been used extensively to identify *Aedes* and *Culex* mosquito species and to perform evolutionary analyses. The universal forward primer LCO1490 (5′-GGT CAA CAA ATC ATA AAG ATA TTG G-3′) and reverse primer HC02198 (5′-TAA ACT TCA GGG TGA CCA AAA AAT CA-3′) described by Folmer et al. [23] were used to amplify a 710 base-pair amplicon of *cox*1 for all *Aedes* species collected. PCR was performed on samples with greater than 20 ng/μl of DNA using the New England biolabs Quick-Load Taq 2× Master Mix (New England Biolabs, Ipswitch, Massachusetts, USA) following the manufacturer’s instructions. The following PCR conditions were used for amplification: an initial denaturing cycle for 2 min at 95 °C, 30 cycles of melting 95 °C for 25 s, annealing at 57 °C for 37 s (with annealing temperature decreasing by 0.4 °C per cycle), extension at 72 °C for 1 min 30 s, followed by an additional 4 cycles with denaturing at 95 °C for 25 s, annealing at 45 °C for 30 s and decreasing by 0.4 °C per cycle, extension at 72 °C for 1 min and 30 s and finished with a final extension step at 72 °C for 10 min. For samples with DNA concentrations beneath the cut-off, RT-PCR was performed on isolated RNA. All RT-PCR reactions were performed by following the recommended protocol of the Roche Titan one-tube RT-PCR system (Roche, Indianapolis, Indiana, USA) with the primers described above. The following conditions were used for all RT-PCR reactions: 1 RT cycle at 45 °C for 45 min followed by 1 denaturing cycle at 94 °C for 4 min, annealing at 45 °C for 1 min, extension at 68 °C for 1 min, followed by 3 cycles of denaturing at 94 °C for 20 s, annealing at 45 °C for 1 min, extension at 68 °C for 1 min, followed by 10 cycles of denaturing at 94 °C for 20 s, annealing at 50 °C for 30 s, extension at 68 °C for 1 min, followed by 16 cycles of denaturing at 94 °C for 20 s, annealing at 50 °C for 30 s, extension at 68 °C for 1 min and increasing by 20 s per cycle and finishing with one extension at 68 °C for 5 min.

All PCR and RT-PCR products were visualized on a 1.5% agarose gel and amplified products were purified using the High Pure PCR product purification kit (Roche) following the manufacturer’s recommended protocol. Following purification, samples were sent for sequencing using both forward and reverse primers (Eurofins genomics, Huntsville, Alabama).

### Sequence alignment and phylogeny reconstruction

Forward and reverse *cox*1 sequences were reviewed for quality and if both reads were successful then these were combined in a single contig; if only one of the two was available then this read was used. The sequences were trimmed and compared using BLAST against the NCBI database. BLAST results with the highest max score were compared to pool sequences and results with a minimum sequence identity ≥ 98% were considered conspecific and sequences with sequence identity > 80% were considered congeners, following the cutoffs laid out by Wang et al. [24].

To review the relatedness of BLAST results to pool sequences, a phylogeny was reconstructed from an alignment of select *Aedes* pool sequences and reference sequences from a wide geographic range retrieved from GenBank. Representative sequences were used for sequences that had 100% sequence identity to each other in order to simplify the phylogeny. All representative sequences and references were aligned using ClustalW and a maximum likelihood phylogeny was created using the Tamura-Nei parameter model and 500 bootstrap replicates. All sequence and phylogenetic analyses were conducted using Geneious version 9.1.4 [25].

### Statistical analyses

Mosquito abundance in different land covers (*n* = 5 sites/land cover) was tested for normality and then compared using a one-way ANOVA followed by a Tukey-Kramer *post-hoc* test to reveal specific differences among land cover types. The proportion of a particular genus or species in two different time periods or two different land covers or groups of land covers was compared using a Fisher’s exact test. Finally, the number of sites that were positive or negative for viral detection in mosquito pools was compared with a Fisher’s exact test, using exceptions described by Freeman & Halton [26].

## Results

### Climate during the 2013–2014 sampling period

Although temperature remained relatively consistent between the two sampling periods (Fig. 2), the monthly average precipitation was higher during the 2013 sampling effort compared to 2014, as expected due to seasonal changes in rainfall.

### Mosquito diversity in the 2013 and 2014 collections

**Fig. 3:**
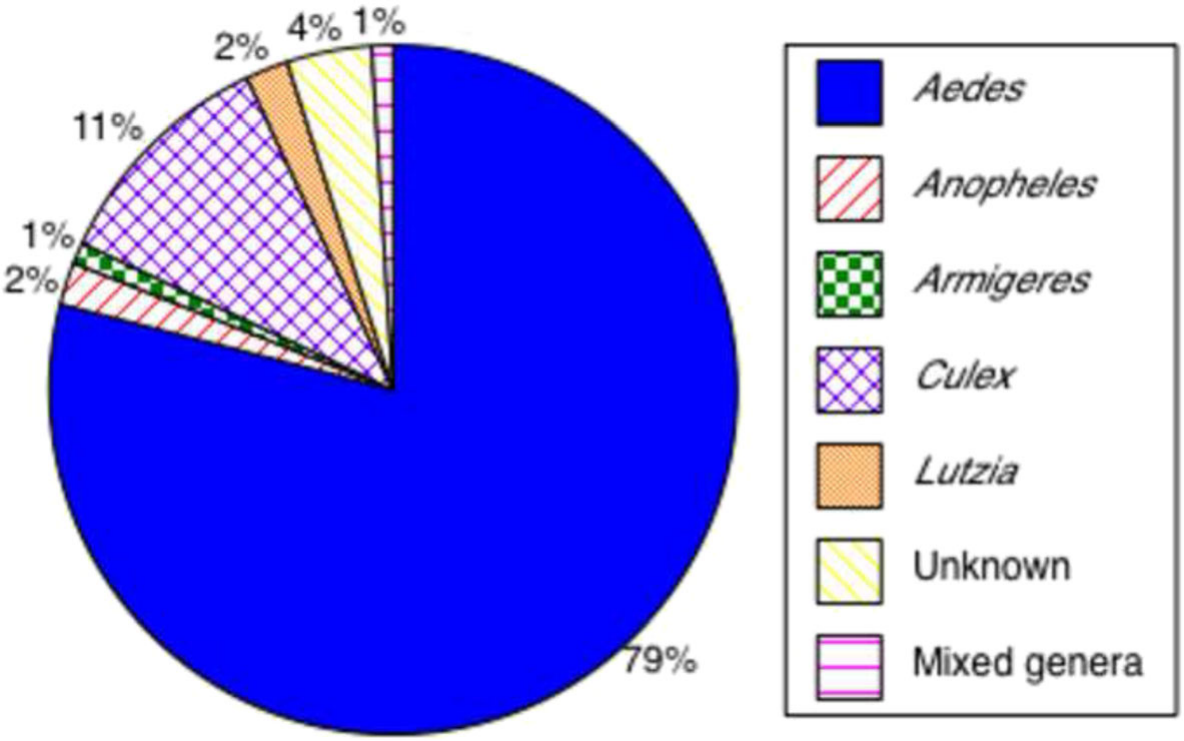
The proportion of mosquito genera collected from 15 sampling sites each in homestead, agricultural and forest land covers in 2013 and 2014 combined. A total of 9 sites (3 per land cover) were sampled between September to December of 2013 and a total of 6 sites between June to July 2014. The mixed genera group includes *Coquillettidia*, *Mansonia*, *Toxorhynchites*, *Uranotaenia* and *Zeugnomyia* which were collected at frequencies of < 1% over the two sampling years

A total of 2164 mosquitoes were collected over the two field seasons, 1201 in 2013 and 963 in 2014, from 5 sites each in homestead, agriculture and forest land covers. Fifty-seven percent were female. All 2164 mosquitoes were pooled into 328 total pools. Based on morphological and molecular identification, the mosquitoes collected belong to 10 genera (Fig. 3). The relative frequencies of genera collected remained consistent over the two years with *Aedes* comprising 79.5% of mosquitoes collected in 2013 and 78.6% in 2014. *Culex* was the second most common taxonomic group, constituting 7.6% of specimens in 2013 and 16.1% in 2014 (Fig. 3). The remaining genera, *Anopheles*, *Armigeres*, *Coquillettidia*, *Lutzia*, *Mansonia*, *Toxorhynchites*, *Uranotaenia* and *Zeugnomyia* were collected at low frequencies, ≤ 4%, over the two sampling years. Mosquitoes that were unidentifiable, or unknown, accounted for 4.1 and 3.1% of the collection, respectively, in the two years. The combined frequencies of the genera collected for 2013 and 2014 are represented in Fig. 3.

### Land cover change and mosquito diversity in the 2016 collection

Of the 15 sites sampled in 2013 and 2014, 2 forests and 1 farm had been altered in 2016. The forests were cleared using the slash and burn method and the farm was converted from solely black pepper to mixed agriculture, demonstrating the quickly shifting landscape in this area. A total of 283 mosquitoes were collected from all sites in 2016, which now included 3 forest sites, 5 agricultural sites, 5 homestead sites, and 1 site each in two new land cover categories: highly degraded forest and barren. Of this collection, 93.2% were *Aedes* and the remaining 6.7% was comprised of *Armigeres*, *Coquillettidia*, *Culex* and *Uranotaenia.* To test whether alteration to sampling sites effected the proportion of *Aedes* sampled in 2016, we compared the proportion of *Aedes* in the total collection of mosquitoes, sampled in 2013 and 2014 to the proportion sampled in 2016. The proportion of *Aedes* collected in the 2016 resampling was significantly greater than in previous sampled years, 2013 and 2014 combined, before the alteration of the sampling sites (*N* = 2447, *P* = < 0.0001). This difference persisted even after excluding the altered sites from the 2016 resampling (*N* = 2437, *P* < 0.0001). We then compared the proportion of *Aedes* sampled in 2013 and 2014 to the proportion sampled in 2016 at each of the 3 altered sites. A total of 179 *Aedes* mosquitoes were sampled at forest site, FSTAT in 2013 and 2014, prior to its conversion to barren land; however, in 2016, no mosquitoes were collected at this site. The proportion of *Aedes* sampled at forest site FSTST (39% of 74), was significantly lower than from the same site after conversion to highly degraded forest in 2016 (86% of 7) (*N* = 81, *P* = 0.04. Finally, the proportion of *Aedes* sampled at agriculture site, FRMCO was higher before (100% of 80) than after (67% of 3) conversion from black pepper monoculture to mixed agriculture, however the extremely small sample size of the post-conversion collection precluded statistical analysis.

### Mosquito DNA barcoding

**Fig. 4:**
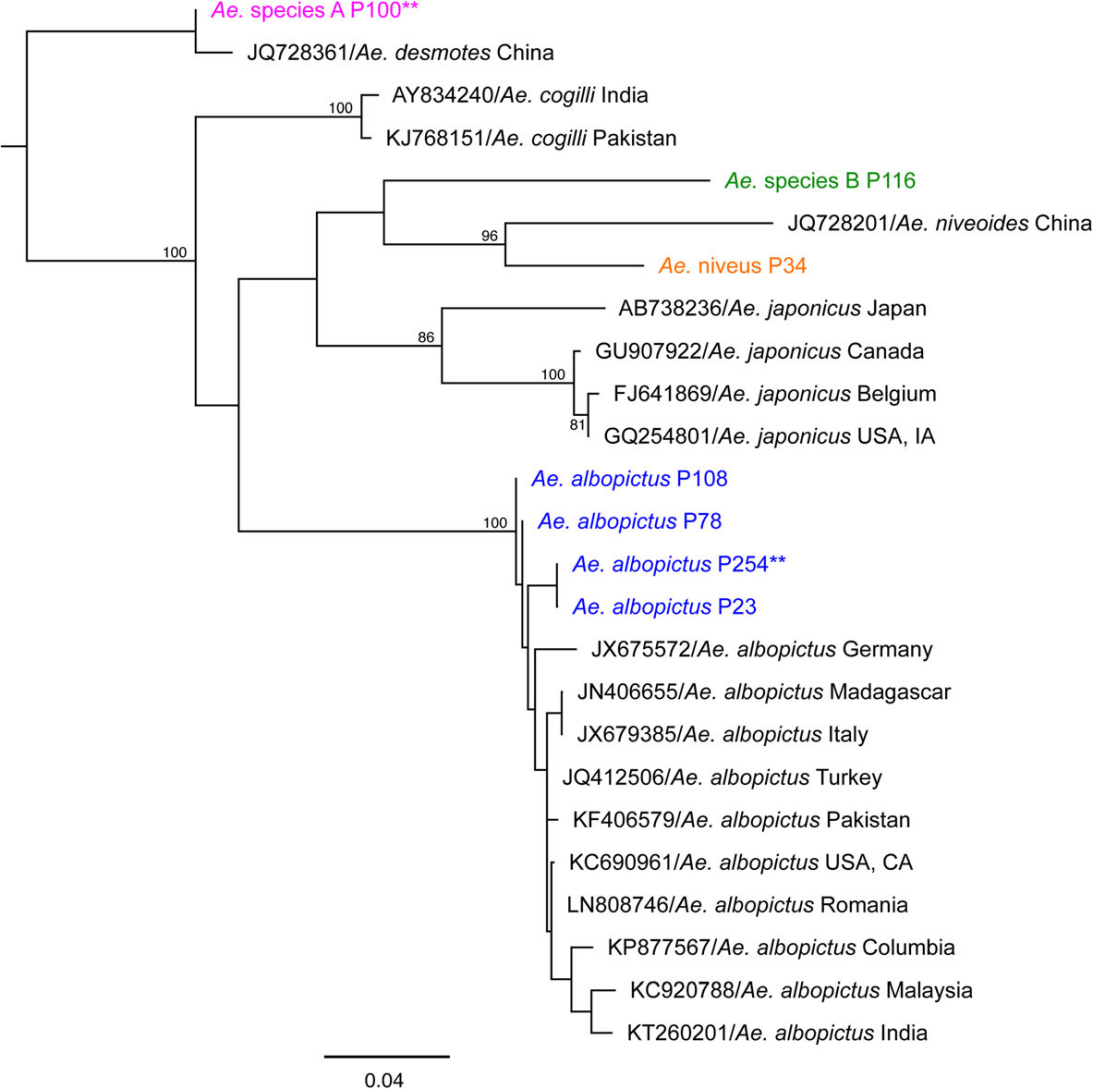
Maximum likelihood phylogeny of representative *Ae. albopictus, Ae. niveus*, *Aedes* sp. A and *Aedes* sp. B sequences from Sarawak and reference sequences from NCBI. Bootstrap values greater than 70% included. See text for further description of *Aedes* sp. A and *Aedes* sp. B. NCBI sequences are named by: accession number/genus_species_country of collection. Sequences generated from pools collected in Sarawak are named by: Genus_species_pool number (e.g. P85) and are shown in colored text. Representatives of multiple *Ae. albopictus* sequences (*n* = 41) and *Aedes* sp. A (*n* = 18) with 100% identity are designated by **

While studies of the ecology of sylvatic mosquitoes in southeast Asia have generally relied exclusively on morphological identification, e.g. [27–30], we felt that in a biodiversity hotspot like Borneo it would also be prudent to barcode specimens. DNA or RNA was extracted from 138 of 193 pools morphologically identified as *Aedes* and subjected to DNA barcoding. A total of 122 of these pools had been identified as *Ae. albopictus* based on morphology; given the high morphological similarity in these individuals, we limited barcoding to 44 pools, which together included 498 mosquitoes. All of these samples, which covered all three land cover types, homestead, forest and agriculture, had between 98 and 99% sequence identity to *Ae. albopictus* sequences when BLAST against the NCBI database (Additional file 1: Table S1). When sequences from all 44 *Ae. albopictus* pools were aligned, the average percent sequence identity for all pairwise comparisons was 99.9%, indicating a high degree of genetic similarity between *Ae. albopictus* populations in the study area. When representative *Ae. albopictus* pool sequences were phylogenetically compared to reference sequences from GenBank, all of the morphologically identified *Ae. albopictus* pools migrated together and showed a close relationship to reference *Ae. albopictus* sequences (Fig. 4).

Of 8 pools of *Ae. niveus* collected, DNA or RNA was successfully amplified from 1 pool, which contained a single mosquito. This sequence did not return a match when BLAST against the NCBI database; an extensive search revealed that no *cox*1 sequences of *Ae. niveus* have been uploaded to GenBank. *Aedes niveus* is a highly distinctive group and we have high confidence in our morphological identification; moreover, it has previously been reported in Sarawak by Macdonald et al. in 1965 [28]. One *cox*1 sequence of an unconfirmed *Ae. niveoides* isolate from China is available in the NCBI database. *Aedes niveoides* is a species in the *niveus* complex that was also identified in Sarawak by MacDonald et al. [28]. A pairwise comparison of this sequence with the *Ae. niveus* sequence indicates 90.2% identity, indicating the two species to be congeners. Our phylogenetic comparison of representative *Aedes* sequences and NCBI references also shows this close relationship between the *Ae. niveus* collected in Sarawak and the *Ae. niveoides* isolate from China (Fig. 4). Thus we here report the *cox*1 sequence of *Ae. niveus* spp. for the first time.

A total of 275 morphologically identical *Aedes* that we were unable to identify to species levels were designated *Aedes* sp. A. The average percent sequence identity among *cox*1 sequences from 18 pools of *Aedes* sp. A, which together included 227 mosquitoes, was 100%, confirming that they are a single species. A BLAST search with these sequences returned a result of *Ae. cogilli* with between 89 and 91% sequence identity (Additional file 1: Table S1). Currently, *Ae. cogilli* has only been described on the Indian subcontinent; however, it is related to the *Aedes niveus* species complex [31]. A phylogeny of representative *Aedes* sp. A and reference *Ae. cogilli* sequences further supports this description, as the collected mosquito pools are more closely related to other *Aedes* sp. A sampled in Sarawak than reference sequences from Pakistan and India (Fig. 4). However, in light of new mosquito collections under way in Sarawak from 2016 and the advancement of available keys from Sarawak, *Aedes* sp. A has now been morphologically identified as *Ae. desmotes*. The average percent sequence identity between an unconfirmed *Ae. desmotes* reference sequence retrieved from GenBank and the *Aedes* sp. A sequences was 94.9% which ranks these specimens as only congeners. However, the reconstructed phylogeny supports this morphological identification over *Ae. cogilli* since the representative *Aedes* sp. A sequences migrate with the reference *Ae. desmotes* sequence from NCBI. Sequencing of other regions of the genome is needed to adequately identify *Aedes* sp. A. A second group of 6 morphologically identical mosquitoes that we were unable to identify were designated *Aedes* sp. B. The *cox*1 sequence from one pool of this species, which included 2 mosquitoes in total, returned a BLAST result of *Aedes japonicus* with 88% sequence identity (Additional file 1: Table S1). Thus this species is closely related to *Ae. japonicus*, a medically important vector of WNV, albeit not a species that has been documented in Borneo [7, 32]. Phylogenetically, this species is more closely related to the *Ae. niveus* specimen collected in Sarawak than to reference *Ae. japonicus* sequences (Fig. 4).

Two groups of morphologically similar *Aedes* species remain unidentified and have been designated as *Aedes* sp. C, which includes 14 mosquitoes in total, and *Aedes* sp. D, which includes 3 mosquitoes in total. Unfortunately, a PCR product was not obtained for either species group using the primers described and therefore a molecular identification for these species could not be obtained. Although these species groups were not identified *via* barcoding, they were nonetheless included in the rarefaction analysis described below.

### Mosquito abundance and distribution in the 2013 and 2014 collection

**Fig. 5:**
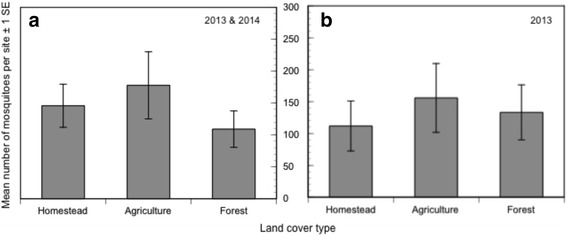
The mean number of mosquitoes and standard error of all species collected per site for each land cover. **a** Data from 2013 and 2014 combined. **b** Data from 2013 alone. There was no significant difference in the mean number of mosquitoes among land covers in either case; see text for statistical analysis

There was no significant difference in the total number of mosquitoes collected among the three land cover types (Fig. 5) (*F*_(2,12)_ = 0.75, *P* = 0.49). Since it was dry prior to sampling in 2014 (Fig. 2), we also analyzed the data from 2013 only (3 sites per land cover); in this comparison also there was no significant difference in the total number of mosquitoes collected among land cover types (Fig. 5) (*F*_(2,6)_ = 0.23, *P* = 0.80). Although there was more limited sampling in 2016, in line with our previous findings, there was no significant difference in the total number of mosquitoes collected among land cover types (*F*_(2,12)_ = 0.23, *P* = 0.13).

**Fig. 6:**
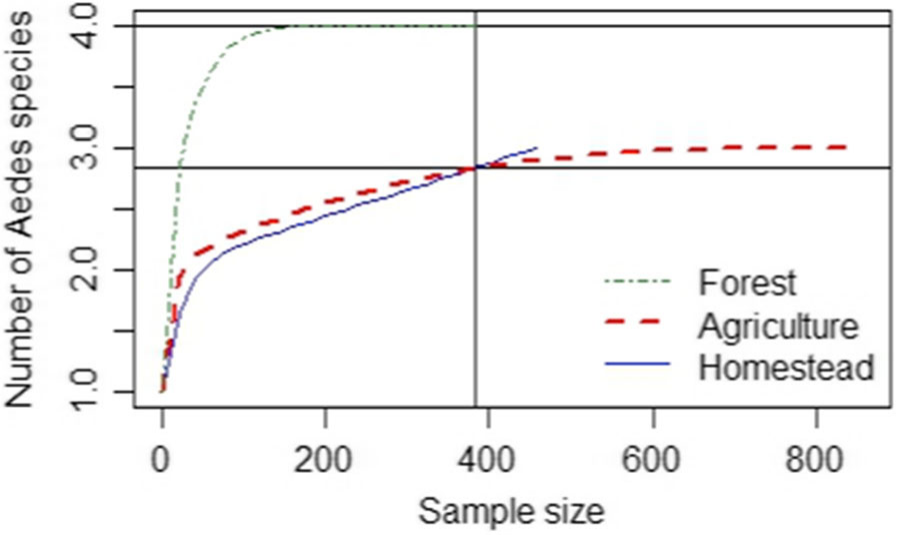
Rarefaction curves showing the diversity of *Aedes* species sampled in three land cover types: homestead, agriculture and forest. The sampling effort in both homestead and agriculture was sufficient in explaining the richness of *Aedes* species present in these land cover types; however, the total diversity of *Aedes* present in forests was not adequately sampled by our efforts

To test whether our sampling efforts were intensive enough to explain the richness of *Aedes* species in all three land cover types, we performed a rarefaction analysis on the total number of *Aedes* collected in all homestead, agriculture and forest sites. This rarefaction analysis indicates that much of the *Aedes* species diversity in the forests remain to be discovered; however, our sampling of agricultural and homestead sites detected most of the *Aedes* species diversity in these land cover types for this area (Fig. 6).

Sampling in 2016 was too limited for statistical analysis.

### Abundance and distribution of target *Aedes* vectors

**Fig. 7:**
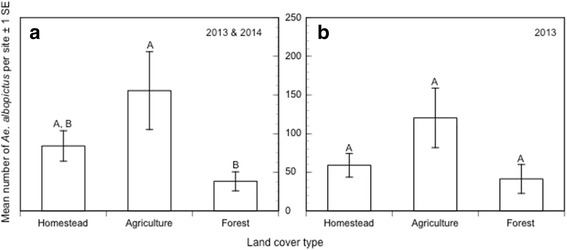
The mean number and standard error of *Ae. albopictus* collected per site in 2013 and 2014 combined (**a**) and in 2013 only (**b**). *Aedes albopictus* was significantly more abundant in agriculture than forest when data from 2013 and 2014 were combined. The statistical analysis of these data is described in the text; significant differences derived from a Tukey-HSD *post-hoc* test are indicated by different letters above bars

The number of *Ae. albopictus* collected among the three land cover types in 2013 and 2014 (Fig. 7) did differ significantly (*F*_(2,12)_ = 4.08, *P* = 0.04); more *Ae. albopictus* were collected in agricultural sites than forests. When the analysis was limited to just 2013, this difference remained marginally significant (*F*_(2,6)_ = 4.96, *P* = 0.05) and *Ae. albopictus* showed a tendency to be more abundant in agricultural sites. We did not analyze the 2014 data by itself as only 2 sites per land cover were sampled in 2014. Counter to our previous findings in 2013 and 2014, there was no significant difference in the mean number of *Ae. albopictus* collected among the three land cover types in 2016 when the altered sites were excluded (mean abundance, HOM = 8.0 ± 8.2, FRM = 41.5 ± 9.1, FST = 15.0 ± 10.5; *F*_(2,9)_ = 3.95, *P* = 0.06). Additionally, there was no significant difference in the percent of *Ae. albopictus* collected among *Aedes* mosquitoes at forest site FSTST, before (45%) and after (33%) conversion to highly degraded forest (*N* = 35, *P* = 0.68).

As only 14 *Ae. niveus* were collected in 2013 and 2014, and indeed all of them were collected in 2013, it was not possible to use ANOVA to compare the abundance of this species in different land covers. Instead, a Fisher’s exact test was used to compare the total number of individuals of *Ae. niveus* and *Ae. albopictus* collected in forest to other land cover types (agriculture plus homesteads). Agriculture and homestead were combined in this analysis due to the very small number of *Ae. niveus* collected in these two land cover types. When the 2013 and 2014 data were combined, the proportion of *Ae. niveus* collected in forests was higher than the proportion of *Ae. albopictus* collected in forests (*N* = 1398, *P* < 0.0001) (Table 2). Since no *Ae. niveus* were collected in 2014, the analysis was repeated using only the 2013 data, and the same overall pattern was detected at a similar level of significance (*N* = 691, *P* < 0.0001). Because of the small number of *Ae. niveus* collected outside of forests (*N* = 3), it was not possible to test whether they showed a preference for forest edge when found in other land cover types. A single *Ae. niveus* was collected during resampling in 2016. Contrary to expectations, this individual was collected at a homestead, which is embedded in a mixed plantation with a relatively high canopy; it is the first *Ae. niveus* specimen we have collected in this land cover type.

**Table 2:** The number of *Ae. albopictus* and *Ae. niveus* mosquitoes collected in non-forest, homestead and agriculture, and forest land cover types in 2013

Species	*Ae. niveus*	*Ae. albopictus*	Total
Land cover	Number (% of all collected)	Number (% of all collected)	
Non-forest	3 (21)	557 (82)	560
Forest	11 (79)	120 (18)	131
Total	14 (100)	677 (100)	

The proportion of *Ae. niveus* collected in forests compared to non-forest sites was greater than the proportion of *Ae. albopictus* collected in forests compared to non-forest sites (Fisher’s exact test, *N* = 691, *P* < 0.0001)

### Virus detection

**Fig. 8:**
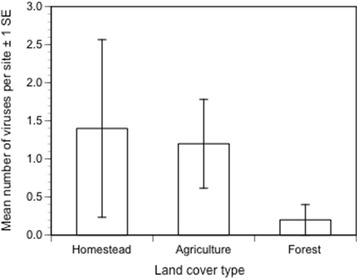
The mean number and standard error of viruses detected per site from *Aedes* mosquito pools collected in 2013 and 2014 combined

Cell culture for arbovirus isolation was performed on 120 *Aedes* pools collected in 2013, including 70 *Ae. albopictus*, 8 *Ae. niveus,* 34 *Aedes* sp. A, 3 *Aedes* sp. B, 3 pools of *Aedes* sp. C and 2 pools of *Aedes* sp. D, and 73 *Aedes* pools collected in 2014, including 52 *Ae. albopictus,* 11 *Aedes* sp. A and 10 pools of *Aedes* sp. C. No virus isolations were made from *Aedes* mosquito pool homogenates placed into Vero cell culture; therefore, all data is based on genome detection by next generation sequencing (NGS) of C6/36 cell cultures showing CPE. NGS was performed on 14 *Aedes* pools that were CPE-positive in C6/36 cell cultures. Genomes of 14 different viruses were detected in 12 of these pools, which included 11 *Aedes albopictus* pools and 1 unidentified *Aedes* sp. C pool. Two pools of *Ae. albopictus* contained genomes from 2 viruses (Table 3). The majority of viruses detected were insect-only viruses including *Euprosterna elaeasa* virus, *Aedes pseudoscutellaris* reovirus, and the newly described Kampung Karu virus [33] (Table 3). Kampung Karu virus is a flavivirus that was isolated from a pool of *Anopheles tessellatus* during the 2013 sampling of this study and lies within a clade of insect only flaviviruses closely related to other flaviviruses that infect humans including DENV [33]. Viruses were detected from mosquito pools collected in all land cover types; however, the majority of identified viruses were either collected in homestead or agriculture land cover types. There was no difference among homestead, agriculture or forest land cover types in the proportion of sites in which a virus was detected (*N* = 15, *P* = 0.80). The small number of samples in which viruses were detected precluded statistical analysis; however, Fig. 8 shows the mean number of viruses detected per land cover type.

**Table 3:** Virus genomes detected by NGS from 12 *Aedes* mosquito pools collected in Sarawak, Malaysian Borneo in 2013 and 2014

Mosquito species	Land class of collection	Sex (M/F) of mosquitoes in pool	No. of mosquitoes in pool	Viruses detected
*Aedes albopictus*	HOM	M	31	*Euprosterna elaeasa* virus; *Aedes pseudoscutellaris* reovirus
*Aedes albopictus*	HOM	F	11	*Euprosterna elaeasa* virus
*Aedes albopictus*	HOM	F	13	*Euprosterna elaeasa* virus
*Aedes albopictus*	HOM	M	27	Kampung Karu virus
*Aedes albopictus*	HOM	M	1	*Euprosterna elaeasa* virus
*Aedes albopictus*	FRM	M	32	*Euprosterna elaeasa* virus; *Aedes pseudoscutellaris* reovirus
*Aedes albopictus*	FRM	M	30	*Euprosterna elaeasa* virus; *Aedes pseudoscutellaris* reovirus
*Aedes albopictus*	FRM	M	22	*Euprosterna elaeasa* virus
*Aedes albopictus*	HOM	M	8	*Euprosterna elaeasa* virus
*Aedes albopictus*	FRM	F	22	*Euprosterna elaeasa* virus
*Aedes* unknown	FST	F	1	*Euprosterna elaeasa* virus
*Aedes albopictus*	FRM	F	7	*Euprosterna elaeasa* virus

*Abbreviations*: F, female; M, male HOM, homestead FRM, agriculture FST, forest

## Discussion

During Rudnick’s expansive ecological study of sylvatic DENV in peninsular Malaysia, he collected 300 species of mosquito, including the 10 genera we collected in Borneo [6]. In 1965 MacDonald et al. [28] also described collecting all of the genera described above, except for *Toxorhynchites*, in Sarawak during the early 1960’s. MacDonald et al. [28] collected 7 genera (*Aedes*, *Culex*, *Anopheles*, *Armigeres*, *Lutzia*, *Mansonia* and *Uranotaenia*) in both rural and forest sites similar to our sampling locations, while they collected *Coquillettidia* and *Zeugnomyia* only in rural areas [28]. As our effort specifically targeted *Aedes*, it is not surprising that this genus makes up the majority of our samples. To date, about 4% of the total mosquito specimens collected remain to be identified, in part because the majority of available dichotomous keys for southeast Asia focus strictly on mosquito species of medical importance [7, 18, 19].

Our molecular identification using *cox*1 of *Aedes* mosquitoes was able to confirm the morphological identification of *Ae. albopictus* with high confidence. The remaining *Aedes* species, *Ae. niveus*, *Aedes* sp. A and *Aedes* sp. B, did not have their identifications confirmed using BLAST and phylogenetic analyses. This was largely due to the lack of available sequences in the NCBI database. For example, *Ae. niveus* is a species complex including 30 species of mosquito which have mainly been described from collections in Malaysia during the 1950’s [7, 34]. MacDonald et al. [28] reported collections of 4 species belonging to the *niveus* subgroup, including *Ae. niveoides*, *Ae. pexus*, *Ae. pseudoniveus* and *Ae. vanus*, in Sarawak in 1962, primarily in forested areas [28]. Of these, only a single unverified *Ae. niveoides cox*1 sequence is available from GenBank. Nonetheless, our study has taken a significant step forward relative to those studies in the region that rely exclusively on morphological identification, and has resulted in the first submission to GenBank of an *Ae. niveus cox*1 sequence. A more robust molecular identification of mosquito species from this region will require an investigation of multiple gene regions [24, 35–40].

We found that forests contained a more diverse mosquito community compared to agriculture and homesteads sampled in Sarawak, Malaysia. This was not unexpected as the rainforests of Borneo are considered a biodiversity hotspot and Borneo is estimated to be the home of over 400 species of mosquitoes [32]. Thongsripong et al. [30] found similar results in Thailand, where both intact and fragmented forests contained the highest diversity of mosquito species and rice fields, the only agricultural land cover type sampled, had the lowest diversity relative to sampling effort.

Our results also support our a priori predictions that *Ae. niveus* would be most abundant in forests and that, compared to *Ae. niveus*, *Ae. albopictus* would be more evenly distributed in all land cover types. Moreover, *Ae. niveus* was collected in the 3 forest sites sampled in 2013 but was not collected at all in 2014, whereas *Ae. albopictus* was collected in all 15 sites sampled, corroborating the greater habitat breadth of *Ae. albopictus*. The absence of *Ae. niveus* in 2014 could be due to typical seasonal patterns of precipitation, which is lower June and July (the sampling months in 2014) than September to December (the sampling months in 2013). Rudnick noted that *Ae. niveus* collections in the 1970’s seemed to peak after periods of consistent rainfall and would dwindle during dry spells [6]. Interestingly, while resampling in 2016, *Ae. niveus* was collected once, but this was within a homestead. The specific site where this individual was collected is surrounded by large, mature fruiting trees as well as several rows of rubber trees. Future studies should compare detection of *Ae. niveus* in homesteads that are or are not surrounded by tall fruit or plantation trees. Despite the small sample size, the detection of *Ae. niveus* almost exclusively in forests indicates that this species is likely to transmit sylvatic DENV only to humans who enter forests or forest edges.

An important caveat to these conclusions is that sampling was conducted at ground level only. Of the 422 *Ae. niveus* females collected by Rudnick in 1974, 95.6% were collected in forest canopy traps placed at 75 ft or higher [6]. However, this suggests that extending our collection efforts into the canopy, which we plan to undertake in the future, would only accentuate the differences in the distribution documented here. It is notable that *Ae. niveus* were collected at ground level, as has been previously observed [6], as nearly all of the forest sites used for mosquito collection were also utilized as foraging sites for local people. Thus humans and potentially infected vectors occur in close proximity, a requirement for viral spillover. While we did not directly observe non-human primates, the only known reservoir hosts of sylvatic DENV [6, 8] in this study, many locals reported seeing monkeys often in the forest sites sampled. Thus the final requirement for viral spillover, co-occurrence of the enzootic host with vectors and humans, was also satisfied in these forests.

Our finding that *Ae. albopictus* is more abundant in agricultural fields but is also found commonly in forests supports its potential to act as a bridge vector for sylvatic DENV, as originally proposed by Smith, in both types of land cover [2, 3]. Brant et al. [41] collected mosquitoes in Sabah, Malaysian Borneo using human landing collections during evening periods and found that *Ae. albopictus* was mainly collected in oil palm plantation, a common agricultural crop in Borneo, and was dramatically less abundant in old growth forests and logged forests. In another study in Sabah, Brant et al. [42] quantified mosquito abundance at ground and canopy levels in different forest classifications. They found that *Ae. albopictus*, though sampled infrequently, were exclusively collected at ground level in logged forests compared to canopy [42], lending support that this mosquito would most likely act as a bridge vector at ground level. Most of the agricultural fields in our study area are either accessed by direct movement through forests or are close to forests, placing *Ae. albopictus* in proximity to non-human primates and humans. Indeed, spillover mediated by *Ae. albopictus* may not require entry into the forest, as local people report that monkeys regularly invade agricultural fields and raid crops.

The drastic conversion of three of our sampling locations between 2014 and 2016 demonstrates the shifting landscapes in this area, and with it the shifting mosquito communities. Collections from two of these sites contained a greater percentage of *Aedes* mosquitoes in 2016. These changes suggest that *Aedes* are particularly able to flourish during land cover conversion. However, the third site, FSTAT, was denuded of vegetation and yielded no mosquitoes in 2016, suggesting that *Aedes* cannot persist when vegetation is totally removed.

In the current small sample, we detected no evidence that land cover type affects the frequency of sites in which viruses were detected. Moreover, we did not detect arboviruses known to circulate in this region, particularly DENV and Zika virus [43]. This was not unexpected, as Rudnick only isolated sylvatic DENV once in more than 800,000 mosquitoes [6]. In the future we plan to expand our mosquito collections to include forest patches of different sizes, primary and secondary forests, and greater vertical range within forests to enhance our likelihood of capturing these critical arboviruses.

Although the viruses detected were solely insect-specific viruses (ISV) [33], they may nonetheless play a role in arbovirus replication and transmission. Interestingly, the majority of ISVs described lie within the same family as many arboviruses that infect humans, Flaviviridae [44, 45]. Most ISVs within this family are thought to be long evolved with their insect hosts, as genes associated with these viruses have been incorporated into vector genomes [44, 45]. These factors are likely involved in the ability of ISV’s to influence arbovirus replication and transmission. For example, Bolling et al. [46] experimentally co-infected mosquitoes with *Culex flavivirus* (CxFV), an insect-only virus, and West Nile virus (WNV) and observed an increase in WNV dissemination at 7 days post infection relative to mosquitoes infected with WNV alone [46]. To date the insect-specific viruses detected in these pools of *Ae. albopictus* have not been fully characterized, but it will be important in the future to test their ability to enhance or suppress arbovirus infections.

## Conclusions

Land conversion has been invoked as an important driver of disease emergence, although few specific mechanisms for such an effect have been demonstrated. However, entry into the forests of Borneo, for leisure, research and construction of infrastructure, was associated with four sylvatic DENV spillover events into humans [11–13, 47]. These accounted for the first isolations of sylvatic DENV from humans in Asia and coincide with drastic changes being made to the landscape, primarily the rainforests of Borneo [11]. Despite its small geographic scope, our study offers the first view of the diversity and distribution of sylvatic *Aedes* vectors in Sarawak, a hotspot of host, vector and arbovirus diversity, since the foundational studies of MacDonald et al. [28] and Simpson et al. [47, 48] in this region nearly fifty years ago. Our study indicates that land cover does indeed affect the abundance and distribution of putative sylvatic DENV bridge vectors, *Ae. albopictus* and *Ae. niveus* and may therefore affect transmission of sylvatic DENV to people. Moreover, in light of current events, it is worth noting that Zika virus circulates in Malaysian Borneo [43] and that *Ae. albopictus* is known to be a competent vector for Zika virus [49] as well as sylvatic DENV.

## Additional file

Additional file 1: Table S1. Sequence data for *cox*1 in *Aedes* mosquito pools: pool ID number, morphological identification, specimen sex (M for male and F for female), land class of collection, homestead (HOM), agriculture (FRM), forest (FST), sample size of pool (N), closest BLAST match, % sequence identity to closest BLAST match, accession number of closest BLAST match and accession number of sequence (DOCX 30 kb)
