# Supplementary material for: Abundance and distribution of sylvatic dengue virus vectors in three different land cover types in Sarawak, Malaysian Borneo
Source: Parasit Vectors. 2017 Aug 31;10:406. doi: 10.1186/s13071-017-2341-z (PMC5580228; doi:10.1186/s13071-017-2341-z)
Supplement: Additional file 1: Table S1. — Sequence data for cox1 in Aedes mosquito pools: pool ID number, morphological identification, specimen sex (M for male and F for female), land class of collection, homestead (HOM), agriculture (FRM), forest (FST), sample size of pool (N), closest BLAST match, % sequence identity to closest BLAST match, accession number of closest BLAST match and accession number of sequence (DOCX 30 kb) [file 13071_2017_2341_MOESM1_ESM.docx]

**Additional file 1: Table S1.** CO1 sequence data for *Aedes* mosquito pools: pool ID number, morphological identification for genus and species, specimen sex (M for male and F for female), land class of collection, homestead (HOM), agriculture (FRM), forest (FST), sample size of pool (N), closest BLAST match, % sequence identity to closest BLAST match, accession number of closest BLAST match and accession number of sequence

| **Pool ID** | **Species (by morphology)** | **Sex** | **Land class** | ***n*** | **Closest BLAST match** | **Sequence identity (%)** | **GenBank closest match accession number** | **GenBank accession number** |
| --- | --- | --- | --- | --- | --- | --- | --- | --- |
| 20 | *Ae. albopictus* | F | HOM | 4 | *Ae. albopictus* | 99 | KR349287.1 | KY817523 |
| 21 | *Ae. albopictus* | F | HOM | 6 | *Ae. albopictus* | 99 | JQ388786.1 | KY817524 |
| 23 | *Ae. albopictus* | F | HOM | 12 | *Ae. albopictus* | 99 | KR068634.1 | KY817525 |
| 25 | *Ae. albopictus* | F | HOM | 14 | *Ae. albopictus* | 99 | KR349287.1 | KY817526 |
| 26 | *Ae. albopictus* | M | HOM | 26 | *Ae. albopictus* | 99 | KC690897.1 | KY817527 |
| 27 | *Ae. albopictus* | F | HOM | 6 | *Ae. albopictus* | 99 | KR349287.1 | KY817528 |
| 29 | *Ae. albopictus* | F | HOM | 11 | *Ae. albopictus* | 99 | KR349287.1 | KY817529 |
| 30 | *Ae. albopictus* | F | HOM | 13 | *Ae. albopictus* | 99 | KR349287.1 | KY817530 |
| 31 | *Ae. albopictus* | M | HOM | 3 | *Ae. albopictus* | 99 | KR068634.1 | KY817531 |
| 33 | *Ae. albopictus* | M | FRM | 4 | *Ae. albopictus* | 99 | KR349287.1 | KY817532 |
| 36 | *Ae. albopictus* | F | FRM | 4 | *Ae. albopictus* | 99 | KR349287.1 | KY817533 |
| 37 | *Ae. albopictus* | F | FRM | 3 | *Ae. albopictus* | 100 | KR349287.1 | KY817534 |
| 38 | *Ae. albopictus* | M | FRM | 1 | *Ae. albopictus* | 99 | KR349287.1 | KY817535 |
| 40 | *Ae. albopictus* | M | FRM | 22 | *Ae. albopictus* | 99 | KC690897.1 | KY817536 |
| 42 | *Ae. albopictus* | F | FRM | 6 | *Ae. albopictus* | 99 | KR349287.1 | KY817537 |
| 76 | *Ae. albopictus* | F | HOM | 3 | *Ae. albopictus* | 99 | KR349287.1 | KY817538 |
| 78 | *Ae. albopictus* | F | HOM | 3 | *Ae. albopictus* | 99 | KC690897.1 | KY817539 |
| 79 | *Ae. albopictus* | F | HOM | 0 | *Ae. albopictus* | 99 | KR349287.1 | KY817540 |
| 80 | *Ae. albopictus* | M | HOM | 1 | *Ae. albopictus* | 100 | KX886285.1 | KY817541 |
| 82 | *Ae. albopictus* | F | HOM | 2 | *Ae. albopictus* | 99 | KR349287.1 | KY817542 |
| 83 | *Ae. albopictus* | M | HOM | 8 | *Ae. albopictus* | 99 | KR349287.1 | KY817543 |
| 84 | *Ae. albopictus* | M | FRM | 37 | *Ae. albopictus* | 99 | KR349287.1 | KY817544 |
| 85 | *Ae. albopictus* | F | FRM | 21 | *Ae. albopictus* | 100 | KX886285.1 | KY817545 |
| 89 | *Ae. albopictus* | M | FRM | 27 | *Ae. albopictus* | 99 | KR349287.1 | KY817546 |
| 90 | *Ae. albopictus* | F | FRM | 21 | *Ae. albopictus* | 99 | KR349287.1 | KY817547 |
| 93 | *Ae. albopictus* | F | FRM | 2 | *Ae. albopictus* | 99 | KU522421.1 | KY817548 |
| 103 | *Ae. albopictus* | M | HOM | 8 | *Ae. albopictus* | 100 | KR349287.1 | KY817549 |
| 108 | *Ae. albopictus* | F | FRM | 5 | *Ae. albopictus* | 99 | KR068634.1 | KY817550 |
| 110 | *Ae. albopictus* | M | FRM | 12 | *Ae. albopictus* | 99 | KR349287.1 | KY817551 |
| 111 | *Ae. albopictus* | F | FRM | 1 | *Ae. albopictus* | 100 | KR349287.1 | KY817552 |
| 113 | *Ae. albopictus* | F | HOM | 4 | *Ae. albopictus* | 99 | KR349287.1 | KY817553 |
| 115 | *Ae. albopictus* | F | HOM | 3 | *Ae. albopictus* | 99 | KR349287.1 | KY817554 |
| 118 | *Ae. albopictus* | M | HOM | 8 | *Ae. albopictus* | 99 | KR068634.1 | KY817555 |
| 119 | *Ae. albopictus* | F | FRM | 18 | *Ae. albopictus* | 99 | KR349287.1 | KY817556 |
| 125 | *Ae. albopictus* | F | HOM | 11 | *Ae. albopictus* | 99 | KF406409.1 | KY817557 |
| 128 | *Ae. albopictus* | M | FST | 3 | *Ae. albopictus* | 99 | KR349287.1 | KY817558 |
| 155 | *Ae. albopictus* | M | FST | 4 | *Ae. albopictus* | 100 | KX886285.1 | KY817559 |
| 158 | *Ae. albopictus* | F | FST | 6 | *Ae. albopictus* | 99 | KR349287.1 | KY817560 |
| 162 | *Ae. albopictus* | M | FST | 7 | *Ae. albopictus* | 99 | KR349287.1 | KY817561 |
| 175 | *Ae. albopictus* | M | FST | 24 | *Ae. albopictus* | 99 | KR349287.1 | KY817562 |
| 187 | *Ae. albopictus* | F | FST | 1 | *Ae. albopictus* | 99 | KR817732.1 | KY817563 |
| 253 | *Ae. albopictus* | M | HOM | 19 | *Ae. albopictus* | 99 | KR349287.1 | KY817564 |
| 254 | *Ae. albopictus* | F | HOM | 25 | *Ae. albopictus* | 99 | KR068634.1 | KY817565 |
| 255 | *Ae. albopictus* | F | HOM | 2 | *Ae. albopictus* | 99 | KR349287.1 | KY817566 |
| 87 | *Ae.* sp. A^a^ | F | FRM | 3 | *Ae. cogilli* | 91 | KF406621.1 | KY817568 |
| 99 | *Ae.* sp. A^a^ | F | FRM | 4 | *Ae. cogilli* | 90 | KF406621.1 | KY817569 |
| 100 | *Ae.* sp. A^a^ | M | FRM | 4 | *Ae. cogilli* | 89 | KF406621.1 | KY817570 |
| 105 | *Ae.* sp. A^a^ | F | HOM | 6 | *Ae. cogilli* | 91 | KF406621.1 | KY817571 |
| 106 | *Ae.* sp. A^a^ | M | HOM | 9 | *Ae. cogilli* | 89 | KU543671.1 | KY817572 |
| 114 | *Ae.* sp. A^a^ | F | HOM | 3 | *Ae. cogilli* | 91 | KF406621.1 | KY817573 |
| 132 | *Ae.* sp. A^a^ | F | FST | 7 | *Ae. cogilli* | 89 | KF406621.1 | KY817574 |
| 152 | *Ae.* sp. A^a^ | M | FST | 11 | *Ae. cogilli* | 90 | KF406621.1 | KY817575 |
| 159 | *Ae.* sp. A^a^ | M | FST | 13 | *Ae. cogilli* | 90 | KF406621.1 | KY817576 |
| 161 | *Ae.* sp. A^a^ | F | FST | 5 | *Ae. cogilli* | 89 | KF406621.1 | KY817577 |
| 165 | *Ae.* sp. A^a^ | F | FST | 11 | *Ae. cogilli* | 89 | KF406621.1 | KY817578 |
| 166 | *Ae.* sp. A^a^ | M | FST | 27 | *Ae. cogilli* | 89 | KF406621.1 | KY817579 |
| 171 | *Ae.* sp. A^a^ | F | FST | 3 | *Ae. cogilli* | 89 | KF406621.1 | KY817580 |
| 172 | *Ae.* sp. A^a^ | M | FST | 6 | *Ae. cogilli* | 91 | KF406621.1 | KY817581 |
| 176 | *Ae.* sp. A^a^ | M | FST | 17 | *Ae. cogilli* | 90 | KF406621.1 | KY817582 |
| 183 | *Ae.* sp. A^a^ | F | FST | 19 | *Ae. cogilli* | 90 | KF406621.1 | KY817583 |
| 184 | *Ae.* sp. A^a^ | M | FST | 9 | *Ae. cogilli* | 90 | KF406621.1 | KY817584 |
| 326 | *Ae.* sp. A^a^ | F | FST | 1 | *Ae. cogilli* | 90 | KF406621.1 | KY817585 |
| 116 | *Ae.* sp. B | F | HOM | 2 | *Ae. japonicus* | 88 | KF211505.1 | KY817586 |
| 34 | *Ae. niveus* | F | FRM | 1 | *Ae. niveoides* | 90 | JQ728201.1 | KY817567 |

^a^*Aedes* sp. A was retrospectively identified as *Aedes desmotes*
